# Condition Changes Before and After the Coronavirus Disease 2019 Pandemic in Adolescent Athletes and Development of a Non-Contact Medical Checkup Application

**DOI:** 10.3390/sports13080256

**Published:** 2025-08-04

**Authors:** Hiroaki Kijima, Toyohito Segawa, Kimio Saito, Hiroaki Tsukamoto, Ryota Kimura, Kana Sasaki, Shohei Murata, Kenta Tominaga, Yo Morishita, Yasuhito Asaka, Hidetomo Saito, Naohisa Miyakoshi

**Affiliations:** 1 Department of Orthopedic Surgery, Akita University Graduate School of Medicine, 1-1-1, Hondo, Akita 010-8543, Japanrkimura@med.akita-u.ac.jp (R.K.); miyakosh@doc.med.akita-u.ac.jp (N.M.); 2 Akita Sports, Arthroscopy, and Knee Group (ASAKG), 1-1-1, Hondo, Akita 010-8543, Japan

**Keywords:** COVID-19, mobile health, self-assessment, sports participation, young athletes

## Abstract

During the coronavirus 2019 pandemic, sports activities were restricted, raising concerns about their impact on the physical condition of adolescent athletes, which remained largely unquantified. This study was designed with two primary objectives: first, to precisely quantify and elucidate the differences in the physical condition of adolescent athletes before and after activity restrictions due to the pandemic; and second, to innovatively develop and validate a non-contact medical checkup application. Medical checks were conducted on 563 athletes designated for sports enhancement. Participants were junior high school students aged 13 to 15, and the sample consisted of 315 boys and 248 girls. Furthermore, we developed a smartphone application and compared self-checks using the application with in-person checks by orthopedic surgeons to determine the challenges associated with self-checks. Statistical tests were conducted to determine whether there were statistically significant differences in range of motion and flexibility parameters before and after the pandemic. Additionally, items with discrepancies between values self-entered by athletes using the smartphone application and values measured by specialists were detected, and application updates were performed. Student’s *t*-test was used for continuous variables, whereas the chi-square test was used for other variables. Following the coronavirus 2019 pandemic, athletes were stiffer than during the pre-pandemic period in terms of hip and shoulder joint rotation range of motion and heel–buttock distance. The dominant hip external rotation decreased from 53.8° to 46.8° (*p* = 0.0062); the non-dominant hip external rotation decreased from 53.5° to 48.0° (*p* = 0.0252); the dominant shoulder internal rotation decreased from 62.5° to 54.7° (*p* = 0.0042); external rotation decreased from 97.6° to 93.5° (*p* = 0.0282), and the heel–buttock distance increased from 4.0 cm to 10.4 cm (*p* < 0.0001). The heel–buttock distance and straight leg raising angle measurements differed between the self-check and face-to-face check. Although there are items that cannot be accurately evaluated by self-check, physical condition can be improved with less contact by first conducting a face-to-face evaluation under appropriate guidance and then conducting a self-check. These findings successfully address our primary objectives. Specifically, we demonstrated a significant decline in the physical condition of adolescent athletes following pandemic-related activity restrictions, thereby quantifying their impact. Furthermore, our developed non-contact medical checkup application proved to be a viable tool for monitoring physical condition with reduced contact, although careful consideration of measurable parameters is crucial. This study provides critical insights into the long-term effects of activity restrictions on young athletes and offers a practical solution for health monitoring during infectious disease outbreaks, highlighting the potential for hybrid checkup approaches.

## 1. Introduction

In December 2019, the coronavirus disease 2019 (COVID-19) broke out in Wuhan [1]. Owing to its rapid spread, the World Health Organization declared COVID-19 a pandemic [2] and governments implemented lockdowns [3], forcing the closure of a variety of noncritical operations, such as sports facilities and events, for extended periods [4].

This abrupt transition substantially disrupted sports event calendars and athletes’ training schedules [5,6]. Additionally, the restrictions on outings considerably reduced the likelihood of participating in physical activity [7,8]. Even during non-pandemic periods, 81% of adolescents could not meet the World Health Organization’s physical activity recommendations [9,10]. A survey across 187 countries showed that the average number of steps taken per person decreased by 27.3% within 30 days of the pandemic declaration [11].

Asia, especially China, implemented the most stringent control policies to contain the infection, disproportionately affecting Asian adolescents and students compared with those in other regions [12]. Consequently, adolescents’ physical activity levels decreased during the pandemic owing to restrictions on outdoor activities [13]. School and sports facility closures, discontinuation of extracurricular sports activities, and limited interaction markedly reduced the time and opportunities for many students to exercise. Decreased physical activity levels led to a decline in adolescent physical fitness, including reduced muscle strength and cardiorespiratory fitness [14]. Supporting evidence for this decline in physical fitness has been documented in recent studies. Bianco et al. [15] conducted an observational study on Italian high school students during the pandemic period, comparing those with and without sports programs. Their findings demonstrated that students who were able to continue participating in regular physical activity at school maintained significantly higher levels of muscular strength and vertical jump performance compared to their peers with fewer training opportunities. This study provided concrete and measurable evidence of how systematic suspension of physical activity had a direct impact on youth fitness parameters, reinforcing the concerns about pandemic-related fitness deficits in adolescents. Furthermore, inadequate and unsystematic training without supervision in such a physical state increases the risk of injury [16].

Athletes accustomed to specialized and intensive daily training would have had to train in restricted conditions. Therefore, even when the pandemic restrictions were lifted, the physical condition of athletes often had not reached a level suitable for return to sports [17]. These situations, inherent in in-home training, also reduced training motivation [18], affected the ability to perform sports-specific tasks even in situations where the restrictions had finally ended [19], and increased potential injury risk after the pandemic [20,21]. The potential for problems, particularly when resuming full-scale competition, was therefore a concern [22].

Specifically, while some studies have examined the immediate impact of lockdowns on physical activity levels [11,13,14], there is a notable scarcity of comprehensive longitudinal investigations tracking adolescent athletes’ physical condition from pre-pandemic baselines through restriction periods and into the recovery phase.

In recent years, the rapid advancement of mobile health (mHealth) applications and telehealth technologies has offered innovative solutions for healthcare delivery, particularly during times when in-person interactions are restricted [23]. These digital tools have demonstrated potential in various aspects of health management, including remote monitoring of physical activity, self-assessment of health parameters, and even guided rehabilitation [24]. For adolescent athletes, such non-contact methods could be crucial in maintaining health surveillance and providing appropriate guidance, mitigating risks associated with both reduced activity and unsupervised training, especially during public health crises when traditional medical checkups are challenging [25]. Despite these technological advancements, studies integrating mHealth solutions for long-term monitoring and injury prevention in adolescent athletes specifically affected by pandemic-related restrictions are still in their nascent stages, with a critical lack of data on their practical efficacy and alignment with traditional assessments.

However, research on the long-term effects of physical activity restrictions during the COVID-19 pandemic on the condition of adolescent athletes, as well as the extent of recovery from these effects following the stoppage of restrictions, remains limited. In essence, there is no clear data on whether pre-pandemic sport injury prevention strategies remain effective under current conditions despite the resumption of sports activities with new safety measures.

Based on these premises, we hypothesized that (1) adolescent athletes would exhibit significantly reduced range of motion and flexibility parameters following the COVID-19 pandemic-induced activity restrictions compared to pre-pandemic levels, and that (2) a newly developed non-contact medical checkup application would largely align with in-person assessments by orthopedic surgeons for key physical parameters, thereby serving as a practical tool for remote monitoring, although some specific measurements might show discrepancies.

Therefore, in this study, our primary objectives were twofold: first, to precisely quantify and elucidate the differences in the physical condition of adolescent athletes before and after the COVID-19 pandemic-induced activity restrictions; and second, to innovatively develop and validate a non-contact medical checkup method suitable for use during an infectious disease outbreak. Despite global efforts to prevent a pandemic in the future, a reliable non-contact medical checkup method and a remote training support system based on its results could contribute to the sport injury prevention system for the next generation of adolescent athletes in similar emergency situations.

## 2. Materials and Methods

### 2.1. Ethical Approval and Informed Consent

This study was approved by the Institutional Review Board of Akita University (Certified Clinical Research Review Board; approval number 1703; date of approval: March 2017). Informed consent was obtained from the parents/guardians of the participants before they participated in the study. This study utilized data from an established community-based medical screening program that has been conducted by the Akita Prefectural Sports Association since 2013. The program involves regular medical checkups for local adolescent athletes during their growth period, with local physicians, physical therapists, and nurses providing individualized advice based on the results. As this study analyzed data from participants in this existing screening program rather than conducting a clinical trial with specific interventions, clinical trial registration was not required.

### 2.2. Study Design and Setting

This cross-sectional observational study was carried out between 1 June 2013 and 30 November 2022. The specific medical checkup methods used in this study have been previously described [26].

A total of 563 adolescent athletes participated in this cross-sectional study. All participants were certified athletes selected by a single prefectural sport association and designated for sport enhancement by the sports enhancement program of the prefectural sport association between 2013 and 2022. Due to the single-day measurement protocol, all recruited participants completed the assessment, resulting in no exclusions or dropouts. The complete dataset of 563 participants was analyzed. Participants were excluded if they or their families did not consent to participation or were unable to participate in a face-to-face evaluation conducted by orthopedic surgeons and physical therapists.

#### 2.2.1. Questionnaire to Assess Pain

First, participants completed a self-administered questionnaire to assess pain during sport activities and height growth within the past year. Because we only inquired about the presence and location of pain, we did not employ a standardized questionnaire. To eliminate the bias that the examiner conducting the medical checkups could alter their assessment based on the presence or location of pain, we adopted a questionnaire format. Participants who reported pain on the questionnaire were asked to identify the location of their pain. The questionnaire was conducted between 2013 and 2022, and since each athlete was identified strictly by their identification numbers, none was assessed more than once.

#### 2.2.2. Physical Examination

Next, we assessed joint range of motion and laxity of the shoulder, elbow, knee, and hip joints; finger–floor distance; the presence of low back pain during lumbar spine extension; heel–buttock distance; “too many toes” sign [11,12,13]; and straight leg raising angle. These assessments were performed indoors at a public facility in June and November of each year by orthopedic surgeons or physical therapists using goniometers or similar devices. None of the athletes were limited because of pain or disability at the time of examination.

For the elbow and knee range of motion, extension < 0° and flexion < 145° were recorded as the limitations of extension and flexion, respectively. For the shoulder joint, the angles of internal and external rotation in 90° abduction were recorded. For the hip joint, the angles of internal and external rotation in 90° flexion were recorded. Joint laxity was evaluated using the test method developed by Whitehead et al. [27]. For the “too many toes” sign [11,12,13], the examiner determined and recorded how many toes (toes IV and V) were visible on the outside of the lower leg from behind the athlete in the standing position. For the detection of lumbar spondylolysis, the presence of “low back pain on lumbar extension” was checked and recorded as a pain provocation test. Joint range of motion was measured using digital goniometers (Poexit Digital Goniometer 360°, 12-inch model, Poexit Co., Karlsruhe, Germany or similar devices with equivalent specifications, accuracy: ±1°), and distance measurements (finger–floor distance and heel–buttock distance) were performed using measuring tapes (Tape Measure 1.5 m, Model 71013, Shinwa Sokutei Co., Ltd., Sanjo, Japan, or equivalent measuring tapes, accuracy: ±1 mm).

Additionally, we assessed the developmental stage of the tibial tuberosity and the site of pain using ultrasonography. The developmental stages of the tibial tuberosity were classified into six stages to predict growth spurts [18,19]. We measured the elasticity of the quadriceps muscle-tendon junction, only on the side that the athlete perceived as dominant, using ultrasound elastography (ACUSON S2000, Ultrasound Division, Siemens Medical Solutions USA, Inc., Malvern, PA, USA or Canon Aplio i700, Canon Medical Systems Corporation, Otawara, Japan), which quantifies elasticity by measuring the shear wave velocity.

#### 2.2.3. Assessment of Pre- and Post-Pandemic Physical Condition and Statistical Analysis

After obtaining the data from the questionnaire and physical examinations, we compared pre-pandemic data (up to 2019) with post-pandemic data (from 2022) for each category, as well as data from 2022 and 2023 to determine the athletes’ recovery time post-pandemic (Table 1). Statistical analyses were performed using SPSS version 27.0 (IBM Corp., Armonk, NY, USA). Descriptive statistics are presented as mean ± standard deviation for normally distributed continuous variables or median (interquartile range) for non-normally distributed variables. Categorical variables are expressed as frequencies and percentages. Normality of continuous variables was assessed using the Shapiro–Wilk test. We used the chi-squared test for categorical variables expressed as percentages and the Student’s *t*-test for normally distributed continuous variables. For non-normally distributed continuous variables, the Mann–Whitney U test was applied. Statistical significance was set at *p* < 0.05. All statistical tests were two-tailed.

#### 2.2.4. Development of a Smartphone Application for Self-Checkup

Based on the data obtained in this and previous studies [26], we developed a smartphone application (Shin Medical Check version 1.1) for self-checks. This application allowed users to perform medical checks, excluding ultrasound examinations, at the team’s practice site or at home, and with immediate feedback based on the results. The application is a standard iOS smartphone app that does not directly measure range of motion or flexibility. Instead, it serves as a data input platform where users enter values from self-assessments or specialist evaluations. The app analyzes these inputs against historical data to identify areas of declining range of motion or flexibility and provides personalized recommendations, including stretching techniques through in-app instructional videos.

#### 2.2.5. Assessment of the Developed Smartphone Application for Self-Checkup

The results of self-check using the smartphone application were compared, item by item, with those of a medical check conducted by orthopedic surgeons in person for the same athletes. Problems with the non-contact medical check method using the application were also investigated. The athletes were asked to perform a self-check using the app on the day of the face-to-face medical checkup by orthopedic surgeons or before. The application’s self-check results were automatically stored based on the player’s identification number and were reviewed after the in-person medical checkup.

## 3. Results

### 3.1. Participant Characteristics

In this study, we examined 563 junior high school students with an average age of 14 years (range: 13–15 years; 315 boys and 248 girls) who had been selected as certified athletes in their prefecture. Athletic activities included swimming, track and field, football, kendo, judo, fencing, tennis, table tennis, badminton, wrestling, sumo wrestling, karate, shooting, basketball, rugby football, gymnastics, canoeing, softball, handball, alpine skiing, Nordic skiing, ice skating, and rifle shooting. All participants consented to participate in this study, and none were excluded because of pain or other reasons that prevented their physical examination.

### 3.2. Pre- and Post-Pandemic Physical Condition

Bilateral hip external rotation range of motion, shoulder joint internal/external rotation range of motion on the dominant side, and heel–buttock distance were all significantly stiffer in the athletes immediately after the pandemic period than prior to it (Table 1). Among the variables that showed statistically significant differences, the effect size (Cohen’s *d*), its interpretation, and the statistical power (1 − β) were as follows. For the range of hip external rotation (dominant side), the effect size was 0.501, which is interpreted as large, with a statistical power of 0.806. For the non-dominant side, the effect size was 0.392 (medium), and the power was 0.600. For shoulder internal rotation (dominant side), the effect size was 0.511 (large), and the power was 0.822. For shoulder external rotation (dominant side), the effect size was 0.390 (medium), and the power was 0.595. For heel–buttock distance, the effect size was −1.033, which is interpreted as large, with a statistical power of 1.000. These results indicate that most of the significant differences were accompanied by moderate to large effect sizes, and sufficient statistical power (≥0.80) was achieved in many cases.

**Figure 1:**
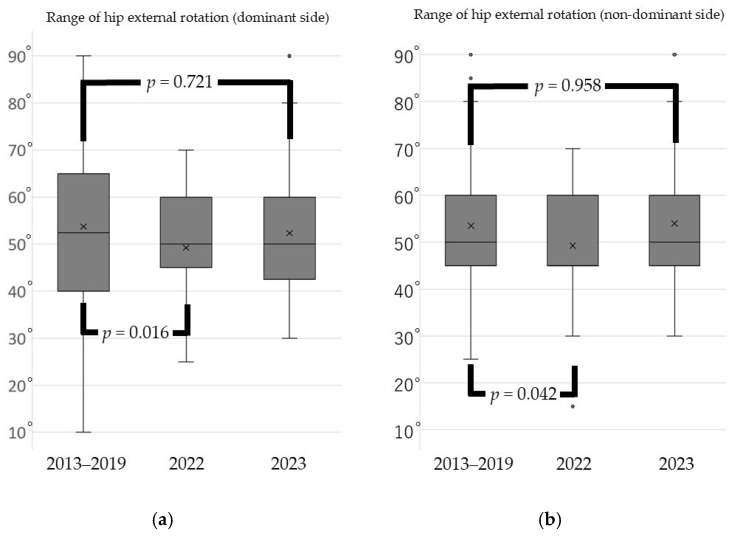
Change in hip external rotation range of motion before the pandemic, immediately after the pandemic, and after the pandemic subsides. (**a**) Range of hip external rotation (dominant side); (**b**) range of hip external rotation (non-dominant side). Box plots show the median, interquartile range, and outliers. Statistical analysis was performed using the Kruskal–Wallis test with Dunn’s test for post-hoc pairwise comparisons. Brackets with *p*-values indicate significant differences between periods (*p* < 0.05).

**Figure 2:**
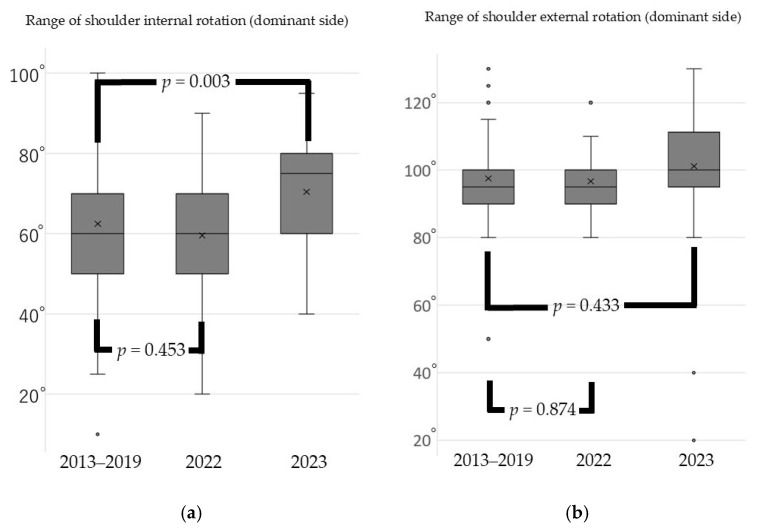
Change in shoulder rotation range of motion before the pandemic, immediately after the pandemic, and after the pandemic had subsided (**a**) Range of shoulder internal rotation (dominant side); (**b**) range of shoulder external rotation (dominant side). Box plots show the median, interquartile range, and outliers. Statistical analysis was performed using the Kruskal–Wallis test with Dunn’s test for post-hoc pairwise comparisons. Brackets with *p*-values indicate significant differences between time periods (*p* < 0.05).

**Figure 3:**
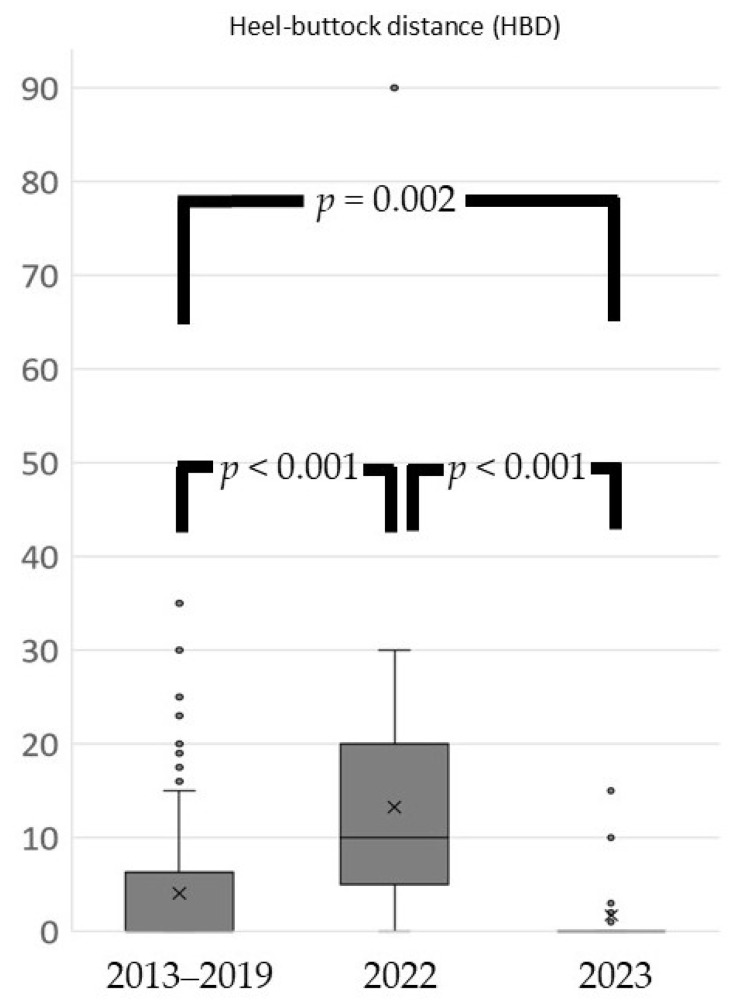
Heel–buttock distance. The heel–buttock distance noticeably increased during the pandemic but improved to surpass pre-pandemic levels within 1 year after the pandemic ended. Box plots show the median, interquartile range, and outliers. Statistical analysis was performed using Kruskal–Wallis test with Dunn’s test for post-hoc pairwise comparisons. Brackets with *p*-values indicate significant differences between periods (*p* < 0.05).

From 2023, Japan’s COVID-19 response policy was revised to align with that for influenza. Consequently, measures such as isolation and movement restrictions for infected individuals and close contacts were eliminated, and treatment at general medical institutions became feasible. During the pandemic, the range of hip external rotation decreased significantly compared with pre-pandemic levels; however, it returned to baseline one year after the pandemic ended (Figure 1). Additional analysis conducted one year after the pandemic revealed that the range of internal rotation of the shoulder joint on the dominant side had not only recovered but also surpassed pre-pandemic levels. In contrast, the range of external rotation of the shoulder joint on the dominant side showed no significant differences across the pre-pandemic, in-pandemic, and post-pandemic periods (Figure 2). The heel–buttock distance increased significantly during the pandemic but improved to exceed pre-pandemic levels within 1 year after the pandemic ended (Figure 3).

### 3.3. Discrepancies Between Physician-Conducted Medical Checkup Results and Self-Checked Results Using the App

Significant discrepancies were found between the application-based self-check and the face-to-face checkup carried out by orthopedic surgeons and physical therapists in the measurements of heel–buttock distance, “too many toes” sign, straight leg raising angle, and ball-and-socket joint range of motion, particularly for the shoulder and hip joints (Table 2).

## 4. Discussion

Few studies have examined how activity restrictions due to the global pandemic have affected the condition of adolescent athletes. We conducted a detailed study to determine the changes in the condition of adolescent athletes before and after the COVID-19 pandemic activity restrictions. The results indicated significant differences in the bilateral hip external rotation range of motion, shoulder joint internal/external rotation range of motion on the dominant side, and heel–buttock distance, all of which were significantly stiffer in the athletes post-pandemic compared with pre-pandemic. Marijančić et al. [28] reported that students with low physical activity levels and prolonged sitting time had imbalanced trunk muscles, worse respiratory function, and poorer quality of life and sleep. Similarly, Goyal [29] demonstrated that sedentary behavior, particularly sitting time >7 h per day, leads to a 5% increase in all-cause mortality and is emerging as a major risk factor for non-communicable diseases among young adults. However, despite these findings on the health impacts of reduced physical activity, there are limited studies specifically examining how prolonged periods of decreased physical activity affect flexibility and range of motion in athletes. Furthermore, these items were no longer significantly different from the survey before the COVID-19 pandemic, only approximately 1 year after nearly all sports activity restrictions were removed. Dietrich et al. [30] reported that early mobilization protocols significantly improve physical outcomes after surgery but noted that the psychological and mental effects of postoperative immobility remain insufficiently studied. Lepers et al. [31] demonstrated that after 12 weeks of detraining, a master triathlete could regain cardiorespiratory fitness with 12 weeks of progressive retraining, though running economy and lean mass remained slightly depressed. However, since most athletes have never experienced detraining periods as long as nearly one year, as occurred during the COVID-19 pandemic, our data provides valuable evidence for understanding recovery from prolonged inactivity.

The observed reduction in range of motion can be explained by the deconditioning effects of prolonged inactivity. Ehresman et al. [32] demonstrated that a sedentary lifestyle contributes to hip flexor tightness and may impair gluteal function in healthy college-aged adults. Their findings showed that targeted stretching interventions could improve hip flexor length, which supports our observation that hip mobility is particularly susceptible to decline during periods of reduced activity. The specific impact on hip external rotation observed in our findings aligns with the notion that prolonged inactivity leads to adaptive changes in hip musculature and surrounding structures, as evidenced by the significant improvements in hip flexibility following structured intervention programs.

We developed a smartphone application for self-checks. The most significant discrepancies in the results between the self-checks using the smartphone application and the face-to-face checkups carried out by medical personnel were observed in the measurements of the heel–buttock distance, “too many toes” sign, straight leg raising angle, and range of motion of the ball-and-socket joints, particularly the shoulder and hip joints. These findings are consistent with previous validation studies of digital health tools. Solheim et al. [33] investigated the agreement between patient-assessed and researcher-assessed measurements of shoulder range of motion, reporting excellent agreement for abduction (Intraclass correlation coefficient [ICC] 0.93) and good agreement for flexion (ICC 0.89), but only moderate agreement for external rotation (ICC 0.72) and internal rotation (kappa 0.63). Their findings confirm that rotational range of motion measurements are particularly challenging for patient self-assessment. However, our study provides novel evidence that flexibility assessments, particularly those requiring passive range of motion, present additional difficulties in remote evaluation, thus contributing to the understanding of digital health tool limitations. If the item was designed to measure the range of motion using a passive technique, the amount of force required to move the examinee’s body could not be standardized, likely increasing the discrepancies between the orthopedic surgeon’s measurement and the application’s self-checks.

Athletes who previously underwent specialized intensive training every day had to adapt to training restrictions during the pandemic. Nevertheless, similar restrictions could arise for individual athletes in the future, even without a pandemic. Furthermore, there was no extensive research on whether sports-specific tasks can be performed once the pandemic subsides and restrictions are lifted. The return of individual athletes from injuries and disorders was widely discussed; however, there is limited research on how quickly adolescent athletes regain their pre-restriction physical condition or the timeline of their recovery, even though there are assumptions that a sudden return to sports after the lockdown may increase injury risks. In this study, we obtained evidence for the above by comparing the results of medical checks on adolescent athletes conducted continuously before the pandemic, immediately after the pandemic, and one year after the restrictions were lifted.

Nevertheless, because face-to-face medical checks could not be conducted during the period when human contact was strongly restricted as a result of the pandemic, we developed a smartphone application that allowed each athlete to conduct personal self-checks. Consequently, this application made it possible to provide a wide range of sports injury prevention measures for adolescent athletes, particularly those who have limited access to frequent medical checkups and expert advice, based on the results of the checkups by sports doctors, even in the absence of a pandemic.

However, this study has some limitations. First, we did not check the same athletes longitudinally but instead compared data from athletes selected by the prefectural sports association for any one year and from year to year; thus, it does not prove that athletes became stiff after COVID-19 because of inactivity during the pandemic. Nevertheless, no study has compared the overall trends in adolescent athletes of the same age during the COVID-19 pandemic, which this study provides valuable evidence. Furthermore, it was impossible to ascertain whether the observed longitudinal changes in the same athletes were attributable to the COVID-19 pandemic or their growth spurts. In this regard, data obtained through the continuous monitoring of athletes of the same age can be considered to hold an even greater value. Second, we were not able to investigate the relationship between the results of the medical checkups in this study and actual sports injuries sustained afterward. Future investigations on the relationship between medical checkup results after the pandemic and the injuries sustained after this study will provide more useful information for the prevention of actual sports injuries. Third, the absence of data during the pandemic, that is, in 2020 and 2021, is also a limitation of this study. To reliably monitor conditions during these periods when in-person checkups are not possible, we developed a smartphone application. However, the results of this study revealed that the accuracy of self-checks using the smartphone application was insufficient and that some items deviated from the in-person check by the orthopedic surgeon. Based on these findings, we updated the application to present the checking method in a video, allowing users to grasp the self-checking method more clearly and use it more accurately. Moreover, the application has been upgraded to present stretching and training methods that are necessary because the checks made using this application are based on the results of in-person medical checkups. Fourth, no formal sample size calculation was performed prior to data collection. Given that data immediately following the COVID-19 pandemic restrictions could only be obtained during a specific time window, we recruited the maximum number of local adolescent athletes available for participation. While this approach may limit the statistical power of our findings, it represents the most comprehensive data collection possible under the unique circumstances of the pandemic period.

Global pandemics such as COVID-19 are rare but impactful, and preventive measures are crucial. Furthermore, even if a new pandemic does not occur, a local influenza endemic may trigger the suspension of sports activities within the affected area for a certain period. Therefore, this study provides useful data when considering the changes in condition that may occur in adolescent athletes who are temporarily unable to train or stretch for sports activities and fitness maintenance due to injury, trauma, or illness. Additionally, research replicating this study on a global scale could provide more reliable global data, making this study a crucial first step in a larger research effort to prevent sports injuries and maintain physical fitness in adolescent athletes.

## 5. Conclusions

This study successfully addressed its primary objectives by quantifying and elucidating the impact of COVID-19 pandemic-induced activity restrictions on the physical condition of adolescent athletes. Our findings demonstrate a significant increase in muscular tightness among adolescent athletes post-pandemic compared to pre-pandemic levels.

Second, we innovatively developed and validated a non-contact medical checkup method designed for use during infectious disease outbreaks. While initial self-assessment through the developed application showed limitations in accurately performing certain assessments, our validation process revealed that combining initial in-person guidance on proper self-assessment techniques with subsequent remote application-based checks effectively improves assessment accuracy and allows for sustained condition management. This iterative development, including updates to the application with video content for self-checks and personalized feedback on stretching and training methods, further enhances the method’s utility and reliability.

These advancements in non-contact medical checkups and remote training support are crucial for establishing a robust sport injury prevention system for future generations of adolescent athletes, particularly in the face of similar emergency situations.

## Figures and Tables

**Table 1:** Changes in the conditions of adolescent athletes before and after the pandemic.

	Before COVID	After COVID	*p*-Value
Restricted finger–floor distance	19.5%	14.5%	0.4940
Laxity of shoulder (dominant side)	82.0%	88.2%	0.3578
Laxity of shoulder (nondominant side)	55.1%	64.7%	0.2700
Restricted range of motion—ankle	22.5%	23.5%	0.8934
Low back pain during lumbar extension	9.83%	8.82%	0.8481
Laxity of finger	25.4%	11.8%	0.0730
Restricted elbow flexion	7.01%	0%	0.1103
Restricted elbow extension	2.08%	2.94%	0.7373
Laxity of elbow extension	45.8%	29.4%	0.0620
Restricted knee flexion	12.9%	11.8%	0.8443
Restricted knee extension	3.04%	2.94%	0.9736
Laxity of knee extension	23.8%	17.6%	0.4142
Straight leg raising angle (dominant side)	75.6°	75.7°	0.9498
Straight leg raising angle (nondominant side)	75.6°	77.1°	0.5710
Range of hip internal rotation (dominant side)	36.8°	34.0°	0.3655
Range of hip internal rotation (nondominant side)	36.8°	36.5°	0.9044
Range of hip external rotation (dominant side)	**53.8** **°**	**46.8** **°**	**0.0062** *
Range of hip external rotation (nondominant side)	**53.5** **°**	**48.0** **°**	**0.0252** *
Range of shoulder internal rotation (dominant side)	**62.5** **°**	**54.7** **°**	**0.0042** *
Range of shoulder internal rotation (nondominant side)	66.4°	61.9°	0.1123
Range of shoulder external rotation (dominant side)	**97.6** **°**	**93.5** **°**	**0.0282** *
Range of shoulder external rotation (nondominant side)	95.9°	92.9°	0.0890
Heel–buttock distance	**4.0 cm**	**10.4 cm**	**<0.0001** *
Growth of body height in 1 year	4.9 cm	4.1 cm	0.4170
Positive rate of “too many toes” sign	22.5%	23.5%	0.889
Reports pain during sports activity	28.6%	32.4%	0.6394

Data are presented as percentages or means. Statistical comparisons between time periods were performed using chi-squared test for categorical variables and Student’s *t*-test for continuous variables. *p*-values < 0.05 were considered statistically significant. * *p* < 0.05.

**Table 2:** Percentage of discrepancies between the results of medical checkups carried out by physicians and those of self-checked using the app.

	Percentage
Restricted finger—floor distance	23.3%
Laxity of shoulder	**33.3%**
Restricted range of motion—ankle	16.7%
Low back pain during lumbar extension	10.0%
Laxity of finger	16.7%
Estimation of elbow flexion angle	0%
Estimation of elbow extension angle	**50.0%**
Estimation of knee flexion angle	13.3%
Estimation of knee extension angle	**33.3%**
Straight leg raising angle	**31.7% (>20** **°** **)**
Range of hip internal rotation	**45.0% (>20** **°** **)**
Range of hip external rotation	25.0% (>20°)
Range of shoulder internal rotation	**83.3% (>20** **°** **)**
Range of shoulder external rotation	**41.7% (>20** **°** **)**
Heel–buttock distance	**50.0% (>5 cm)**
Positive rate of “too many toes” sign	**33.3%**

Data are presented as percentages of cases in which self-assessment differed from physician evaluation.

## Data Availability

The original data presented in the study are openly available in FigShare at https://doi.org/10.6084/m9.figshare.29377442.

## Abbreviations

The following abbreviations are used in this manuscript:

COVID-19	Coronavirus disease 2019
ICC	Intraclass correlation coefficient
